# Workplace violence, psychological capital, and professional identity among Chinese nursing interns: a latent profile and mediation analysis

**DOI:** 10.3389/fpubh.2026.1791963

**Published:** 2026-03-03

**Authors:** Yuqing Liang, Lili Chen, Jie Wang, Dan Su

**Affiliations:** School of Nursing, Anhui Medical University, Hefei, China

**Keywords:** latent profile analysis, mediation analysis, nursing interns, professional identity, psychological capital, workplace violence

## Abstract

**Aims:**

To identify latent professional identity profiles among nursing interns and to examine the influencing mechanisms of workplace violence (WPV) and psychological capital (PsyCap) on these profiles.

**Background:**

Developing a strong professional identity is crucial for nursing interns’ transition to clinical practice and addressing the global nursing shortage. However, professional identity formation varies, and its heterogeneity among interns remains poorly understood. Identifying distinct identity subgroups and clarifying influencing mechanisms can help provide tailored educational support.

**Methods:**

Convenience sampling recruited 313 nursing interns from tertiary hospitals across 9 provinces in China between November 2023 and April 2024. The survey included general information questionnaires, workplace violence scales, professional identity questionnaire, and positive psychological capital questionnaire. Analyses of latent profiles and mediations were performed.

**Results:**

Among 301 participants, latent profile analysis revealed 3 distinct profiles: identity deficient (10.3%), moderate identity (47.2%), and high identity-low autonomy (42.5%). The results showed that self-efficacy, hope, and emotional abuse among nursing interns emerged as significant predictors of the category of professional identity (*p* < 0.001). The mediating effect value of PsyCap between WPV and professional identity was −0.171 (95% CI: −0.286 to −0.086), accounting for 63.10% of the total effect.

**Conclusion:**

The analysis revealed heterogeneity in nursing interns’ professional identities. WPV and PsyCap are key factors influencing this heterogeneity, with PsyCap serving as a partial mediator. Targeted interventions based on identity profiles are recommended, alongside ensuring clinical safety and integrating PsyCap training into education to support the stability of the nursing workforce.

## 1 Introduction

Globally, the shortage of healthcare workers represents a pressing concern for healthcare systems. While the nursing workforce is expanding, a significant deficit persists. The World Health Organization reported a shortage of 5.8 million nurses in 2023, a figure projected to decline yet remain substantial at 4.1 million by 2030 (1). Nursing interns serve as a crucial reserve for the clinical nursing workforce. Thus, their training and career stability are vital to addressing the nursing shortage. In this context, the question of how to effectively cultivate and retain this group, supporting their transition from students to practicing nurses and their professional socialization, has garnered significant attention. Addressing this issue warrants further exploration within nursing education and management to promote their long-term retention.

The stability of the nursing profession is closely linked to the establishment and development of professional identity (2). Professional identity refers to an individual’s positive cognitive and emotional acceptance of their occupation, along with the recognition of its value (3). For nursing interns, it includes positive perceptions of the profession’s value, the professional group, and their own sense of belonging. A well-developed professional identity can foster sustained and stable career behavior. Research indicates that professional identity influences interns’ physical and mental health, academic engagement, and future career choices (4). Those with stronger professional identity typically show better adaptability and retention intent, whereas insufficient identity is often associated with increased stress, diminished practice quality, and compromised career stability (5). The internship phase is a critical period for interns to achieve professional socialization (6). During this period, interns apply their theoretical knowledge to clinical practice, which not only enhances their professional skills and clinical judgment but also familiarizes them with nursing workflows. Through firsthand experience, they internalize professional traits and recognize the value of nursing, thereby potentially deepening their professional identity over time. However, the internship also brings significant challenges. Interns often face multiple pressures, including heavy workloads, strained relationships, skill deficiencies, difficulties in role transition, and career uncertainty (7). Additionally, they may be exposed to workplace violence.

Workplace violence (WPV) encompasses verbal abuse, threats, and physical attacks experienced by staff in work-related settings. It can endanger both physical and mental health, and may threaten professional identity. Research indicated that approximately 8 to 38% of healthcare workers globally have experienced WPV, with this figure rising to as high as 50% among nursing interns in China (8). Compared to experienced nurses, interns may be more vulnerable to violent incidents due to their younger age, limited social experience, and insufficient professional practice skills (9). According to Selye’s stress response theory (10), prolonged exposure to high-intensity stressors depletes an individual’s adaptive capacity and can eventually lead to an exhaustion phase. This phase is associated with impaired physical and mental functioning and may trigger strong avoidance motivation. Thus, repeated WPV exposure could have substantial negative impacts on nursing interns, potentially affecting their physical and mental health, professional identity, and future career planning (11). To analyze the mechanisms through which WPV might influence professional identity, this study adopts social identity theory as the overarching framework (12). This theory suggests that individuals derive a sense of self-worth from their group membership. WPV can be seen as a form of negative intergroup behavior. When interns experience violence in their capacity as nurses, it may cause personal distress, devalue the professional role, and thereby undermine their professional identity.

Social identity theory further suggests that when facing threats to group identity, individuals often need to mobilize internal psychological resources to maintain identity stability (12). Psychological capital (PsyCap), as a key positive psychological resource (13), encompasses four dimensions: self-efficacy, hope, resilience, and optimism. It can provide support for individuals in coping with external threats such as WPV, which may contribute to maintaining occupational stability. Research indicates that PsyCap can help interns promptly regulate negative emotions, alleviate occupational burnout, and support professional growth (14). It has also been associated with enhanced job satisfaction, reduced stress, and lowered turnover tendencies (15). As an internal psychological resource, PsyCap might mitigate the negative impact of WPV on interns’ professional identity. While PsyCap’s mediating role is recognized among nurses (15), interns are at a key identity formation stage. Their social experience, professional competencies, and psychological regulation abilities can differ from practicing nurses. Therefore, the specific mechanisms by which WPV might affect professional identity through PsyCap in this group, as well as the potential heterogeneity within the group, warrant further exploration.

To address this, our study combines latent profile analysis (LPA) with a mediation model. LPA divides the population into subgroups that exhibit internal similarity by examining latent class structures among observed variables (16). It is widely used in psychology, education, and sociological research and is therefore particularly suitable for revealing diverse patterns of professional identity. While existing research has predominantly focused on classifying professional identity itself and analyzing its general influencing factors (17), there has been less systematic examination of the differential mechanisms involving WPV and PsyCap across distinct professional identity profiles. This study aims to address this insufficient aspect in the literature by providing empirical evidence and strategic support for enhancing interns’ professional identity and improving the nursing environment. Based on the above, the following hypotheses are proposed:

H1: Distinct profiles of professional identity can be identified among nursing interns using LPA;

H2: WPV is significantly associated with professional identity;

H3: PsyCap might mediate the relationship between WPV and professional identity.

## 2 Methods

### 2.1 Design

This cross-sectional study investigated latent categories of professional identity among nursing interns in China and their associations with WPV and PsyCap, following the STROBE guidelines.

### 2.2 Participants and sample size

In this research, conducted from November 2023 to April 2024, nursing interns undertaking internships at Grade A Tertiary Hospitals were recruited using convenience sampling. No grade level was specified, as internships usually take place in the final stage of nursing education, whereas postgraduate trainees may have longer clinical exposure. The inclusion criteria were: (1) informed consent and voluntary participation; (2) enrollment in a nursing program with ongoing hospital internship. The exclusion criteria included: (1) questionnaire completion time ≤75 s; (2) contradictory responses; (3) students who self-reported a history of clinically diagnosed psychiatric disorders. A literature review indicated that, from the perspective of model classification accuracy, at least 50 subjects per category are required to ensure the correctness of model selection (18). Assuming 3 to 5 potential categories, a minimum of 250 participants was required. The sample included in this study was therefore considered adequate.

### 2.3 Instruments

#### 2.3.1 Demographic information

The researchers designed a questionnaire on the general information of the subjects, including gender, age, monthly family income, education level, internship duration, etc.

#### 2.3.2 Workplace violence

The Workplace Violence Scale (WVS) was originally developed by Schat and Kelloway (19), and was later adapted by Wang et al. (2006) to the Chinese context to assess the prevalence of WPV in China (20). This scale comprises five key items: physical assault, emotional abuse, threats, verbal sexual harassment, and physical sexual harassment. Frequency is rated on a 4-point scale: 0 (never), 1 (once), 2 (2–3 times), and 3 (≥4 times). The original scale demonstrated good reliability, with a Cronbach’s *α* of 0.88. In the present study, the Cronbach’s *α* was 0.766, which is acceptable for group-level comparisons and consistent with previous use in similar contexts (15).

#### 2.3.3 Professional identity

The professional identity questionnaire for nurse students (PIQNS) (21) was developed by Hao Yufang. The 17-item instrument comprises five dimensions: professional self-concept (6 items), benefits of staying and risks of leaving (4 items), social comparison and self-reflection (3 items), autonomy in career choice (2 items), and social persuasion (2 items). Each item is rated on a 5-point Likert scale, with higher scores indicating stronger professional identity. The original questionnaire shows good reliability, with a Cronbach’s *α* of 0.827 and a split-half reliability of 0.842. In the present study, the Cronbach’s *α* reached 0.958.

#### 2.3.4 Psychological capital

The measurement of PsyCap employs the Chinese version of the Positive Psychological Capital Questionnaire, developed by Zhang Kuo (22) based on Luthans’ theoretical framework (23). The 26-item scale includes four factors: self-efficacy (7 items), resilience (7 items), optimism (6 items), and hope (6 items). Responses are recorded on a 7-point Likert scale ranging from 1 (“Not at all”) to 7 (“Exactly”), with higher total scores indicating greater psychological capital. The original scale showed a Cronbach’s *α* of 0.89, demonstrating good internal consistency. In the present study, the Cronbach’s *α* was 0.945.

### 2.4 Quality control

Before the formal survey, a pilot survey was conducted with 20 nursing interns to verify the questionnaire’s appropriateness. The survey was administered online via Wenjuanxing, a platform that supports link and QR code distribution through social media platforms such as WeChat and QQ. After receiving uniform training, research team members used a standardized Wenjuanxing link to collect data. The questionnaire’s first page outlined the study purpose, instructions, and key notes, emphasizing voluntary and anonymous participation with informed consent. To ensure data quality, respondents were required to answer all items before submission, with a pop-up alert reminding them of any omissions. Each IP address was restricted to one response to prevent duplicates. After data collection, the principal investigator exported responses from the backend, and two team members independently reviewed and excluded non-compliant questionnaires. A total of 313 questionnaires were collected. Participants were recruited from Grade III Class A teaching general hospitals across nine provincial-level regions in China (Anhui, Jiangsu, Zhejiang, Beijing, Guangdong, Shanghai, Shandong, Henan, and Chongqing). Following screening, 301 valid questionnaires were retained, resulting in an effective response rate of 96.17%.

### 2.5 Statistical analysis

Data analyses were conducted using SPSS 26.0 and Mplus 8.3. The analytical procedure was as follows. First, descriptive statistics were computed for demographic variables, presented as frequencies (percentages) for categorical data and means (±standard deviations) for continuous data. Subgroup differences were examined using independent samples *t*-tests. Pearson correlation analysis assessed bivariate relationships among WPV, PsyCap, and professional identity. Second, LPA was performed to identify professional identity subgroups, using the five dimension scores as manifest variables. The optimal model was selected based on fit indices: the Akaike Information Criterion (AIC), Bayesian Information Criterion (BIC), sample-size adjusted BIC (aBIC), entropy, the Lo–Mendell–Rubin adjusted likelihood ratio test (LMRT), and the Bootstrap Likelihood Ratio Test (BLRT). Lower AIC, BIC, and aBIC values indicated better model fit. Entropy values range from 0 to 1, with values closer to 1 denoting higher classification accuracy. A significant *p*-value (<0.05) for both the LMRT and BLRT suggests that the k-class model provides a better fit than the (k-1)-class model. Third, after identifying latent classes, one-way ANOVA was used to compare PsyCap and WPV across profiles. An unordered multinomial logistic regression examined the predictive effects of PsyCap and WPV on profile membership. Finally, the mediating role of PsyCap between WPV and professional identity was tested using the PROCESS macro (Model 4) with 5,000 bootstrap samples (24). All variables were standardized prior to analysis, and a 95% bias-corrected confidence interval excluding zero indicated a significant mediation effect. All tests were two-tailed, with *p* < 0.05 considered statistically significant.

## 3 Results

### 3.1 General information

The study sample consisted of 301 nursing students. The majority were female (261, 86.7%), with a mean age of 20.1 years (SD = 1.42, range: 17–28). Regarding the duration of clinical practice, most students (216, 71.8%) had over 6 months of clinical practice, while others practiced for 3–6 months (73, 24.3%) or under 3 months (11, 3.7%). In terms of educational level, 256 (85.0%) were college-educated, 39 (13.0%) were undergraduate students, and 6 (2.0%) were postgraduates. Concerning average monthly household income per capita, 124 participants (41.2%) reported an income of 3,000–5,000 Chinese yuan (CNY), 82 (27.2%) reported 5,001–10,000 CNY, 56 (18.6%) under 3,000 CNY, and 39 (13.0%) over 10,000 CNY.

### 3.2 Correlation analysis

Harman’s single-factor test was conducted to assess common method bias. The results showed that 11 factors had eigenvalues greater than 1. The first factor accounted for 38.14% of the total variance, which is below the recommended threshold of 40%. Therefore, common method bias was unlikely to be a serious concern in this study. Descriptive statistics and correlations for the main variables are presented in Supplementary Table 1. Means and standard deviations were as follows: WPV (M = 1.09, SD = 2.15), PsyCap (M = 118.96, SD = 21.93), and professional identity (M = 56.71, SD = 12.52). Pearson correlations indicated that WPV negatively correlated with PsyCap (*r* = −0.26, *p* < 0.001), PsyCap positively correlated with professional identity (*r* = 0.70, *p* < 0.001), and WPV was negatively correlated with professional identity (*r* = −0.31, *p* < 0.001). See Supplementary Table 1 for details.

### 3.3 Potential profile analysis results and naming of nursing interns’ professional identity

Starting from the initial model, sequentially establish 1–5 latent category models, as shown in Table 1. Generally, lower AIC, BIC, and aBIC values indicate better model fit. A higher Entropy value closer to 1 suggests clearer classification and a higher probability of accurate assignment. Significant LMR and BLRT values indicate the k-class model fits better than the (k-1)-class model.

**Table 1:** Fit statistics for latent profile models of professional identity.

Model	1	2	3	4	5
AIC	3,662.191	3,090.100	2,695.963	2,366.390	2,207.748
BIC	3,699.262	3,149.414	2,777.519	2,470.189	2,333.790
aBIC	3,667.548	3,098.671	2,707.748	2,381.389	2,225.961
Entropy	–	0.922	0.959	0.977	0.978
BLRT	–	<0.001	<0.001	<0.001	<0.001
LMR	–	<0.001	<0.010	<0.010	0.052
Classification	–	0.55/0.45	0.48/0.10/0.42	0.36/0.10/0.46/0.08	0.04/0.10/0.35/0.43/0.08

As shown in Table 1, AIC, BIC, and aBIC decrease monotonically with the increase in the number of categories. The rate of decrease slows from 2 to 4 categories and becomes more gradual, with all Entropy values remaining above 0.9. Additionally, the LMR value for the 5-category model was not significant, indicating that it did not outperform the 4-category model. Furthermore, considering model accuracy and to avoid misclassification, each subgroup should contain at least 10% of the sample size (25). Therefore, we selected the 3-category model as optimal. The average probability of professional identity attribution for interns in each category is 0.990, 0.960, and 0.983, respectively.

**Figure 1:**
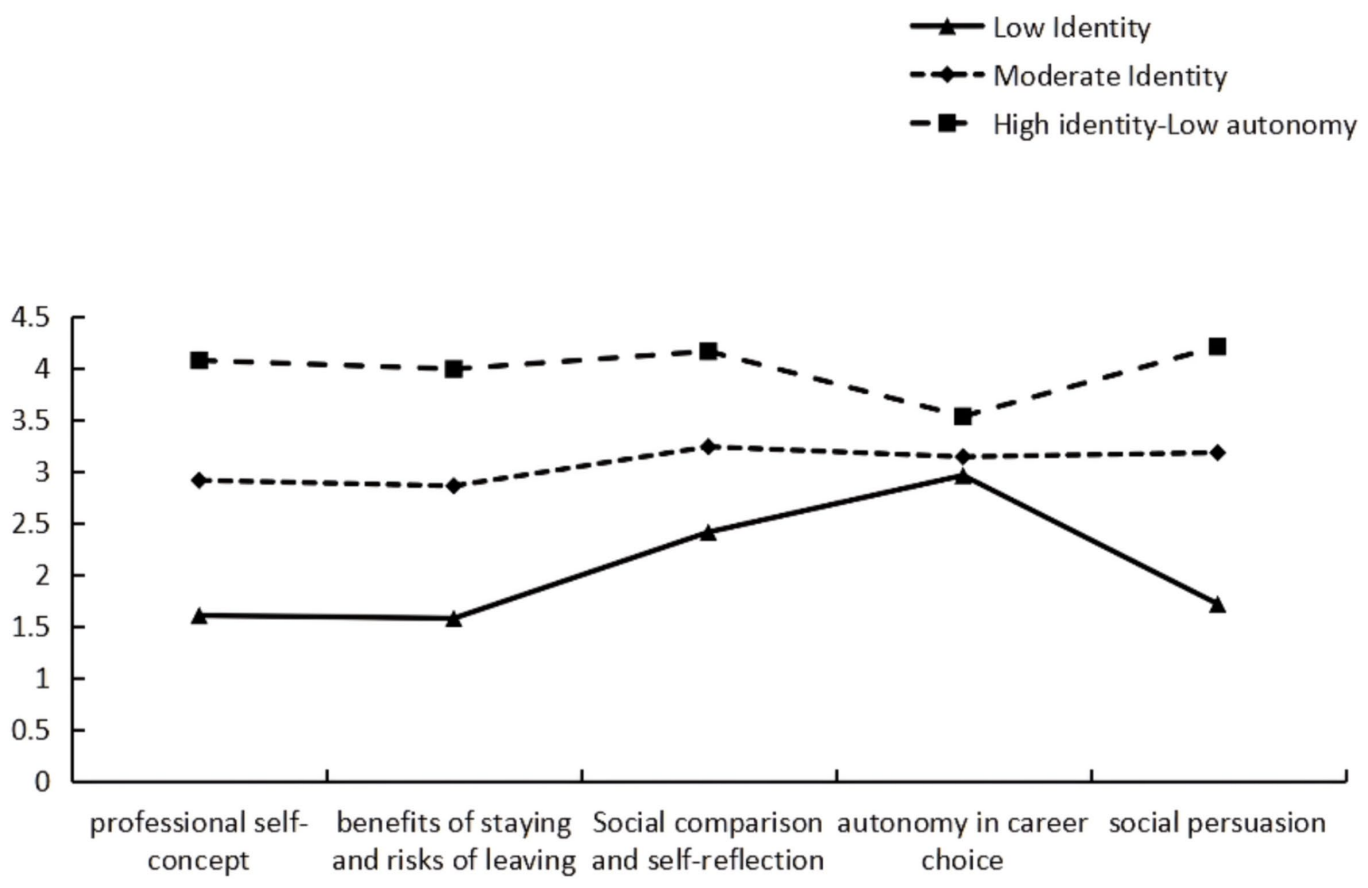
Profiles of professional identity (*n* = 301).

The scores of the three categories of interns on the five dimensions of professional identity are shown in Figure 1. The first category, consisting of 31 nursing interns (10.3%), scored the lowest across all dimensions of professional identity, ranging from 21 to 41 points (out of a total of 85 points). This is particularly evident in the dimensions of professional self-concept, benefits of staying and risks of leaving, as well as social persuasion; hence, it was named the “Low Identity” group. The second category (*n* = 142, 47.2%) had moderate scores (42–59). Its scores were relatively close across all dimensions; therefore, it was labeled the “Moderate Identity” group. The third category (*n* = 128, 42.5%) had high scores overall (59–85). However, autonomy in career choice (noting that this dimension was measured with only two items) was relatively lower than the other dimensions within this profile; therefore, this class was labeled as the “High identity-Low autonomy” group.

### 3.4 Characteristics and differences of nursing interns in WPV and PsyCap across professional identity profiles

Analysis of variance revealed significant differences in all PsyCap dimensions among interns in the professional identity profiles. Significant differences were also found in the emotional abuse and threatening intimidation dimensions of WPV (See Supplementary Table 2). Post-hoc analysis showed the High identity-Low autonomy group scored higher on self-efficacy, hope, optimism, and total PsyCap than the other two groups. This group also reported lower emotional abuse, threats, and total WPV. Moderate identity group scored higher than the low identity group on self-efficacy, resilience, optimism, and total PsyCap. However, there was no significant difference between these two groups in hope, emotional abuse, threats, or total WPV.

### 3.5 Analysis of influencing factors on professional identity profiles of nursing interns

As shown in Table 2, using the low identity group as reference, gender, age, and internship duration were not significant predictors. In terms of education level, interns with bachelor’s or master’s degrees were less likely than those with associate degrees to belong to the moderate identity group or high identity-low autonomy groups. Regarding household monthly income, nursing interns with a monthly household income of 3,000–5,000 CNY were more likely to be in the high identity-low autonomy group than those earning over 10,000 CNY.

**Table 2:** Multiple logistic regression analysis of the related factors on each potential category group.

Variables	LPA-based professional identity class			
	Univariate analysis			
	Moderate identity vs. Low identity		High identity–Low autonomy vs. Low identity	
	OR(95%CI)	*P*	OR(95%CI)	*P*
Age	0.790 (0.617–1.012)	0.062	0.843 (0.661–1.075)	0.168
Gender (male as ref)	0.584 (0.126–2.695)	0.491	0.315 (0.70–1.414)	0.132
Education (associate degree and below as ref)				
Bachelor degree	0.183 (0.074–0.451)	<0.001	0.136 (0.052–0.355)	<0.001
Postgraduate	0.137 (0.018–1.038)	0.054	0.148 (0.020–1.120)	0.064
Internship duration (less than 3 months as ref)				
Three to six months	2.250 (0.200–25.369)	0.512	1.375 (0.130–14.258)	0.791
More than 6 months	0.981 (0.105–9.150)	0.986	0.571 (0.066–4.955)	0.611
Monthly household income (>10,000 CNY as ref)				
<3,000 CNY	1.476 (0.419–5.204)	0.545	1.583 (0.413–6.063)	0.502
3,000–5,000 CNY	1.750 (0.540–5.669)	0.351	4.187 (1.231–14.239)	0.022
5,001–10,000 CNY	1.065 (0.346–3.281)	0.913	1.364 (0.411–4.523)	0.612
Self efficacy	6.763 (1.066–1.232)	<0.001	161.313 (1.365–1.650)	<0.001
Tenacity	1.197 (1.046–1.187)	0.067	1.589 (1.117–1.285)	<0.001
Hope	1.120 (0.993–1.192)	0.001	1.206 (1.367–1.726)	<0.001
Optimistic	1.098 (1.038–1.225)	0.209	1.581 (1.426–1.782)	<0.001
Psychological capital	3.514 (1.020–1.071)	0.070	25.479 (1.115–1.190)	<0.001
Emotional abuse	0.490 (0.352–0.724)	<0.001	0.290 (0.198–0.457)	<0.001
Threaten and intimidate	0.612 (0.371–1.036)	0.074	0.356 (0.187–0.716)	0.003
Work violence	0.870 (0.764–0.989)	<0.050	0.672 (0.571–0.833)	<0.010

Regarding the prediction of group membership, using the low identity group as reference, four PsyCap and WPV factors were significant for the moderate identity group: higher self-efficacy and hope, and lower emotional abuse and workplace violence. For the high identity-low autonomy group, eight factors were significant: higher scores on psychological capital, self-efficacy, resilience, hope, and optimism, and lower emotional abuse, threats, and workplace violence.

### 3.6 Mediation analysis of PsyCap

**Figure 2:**
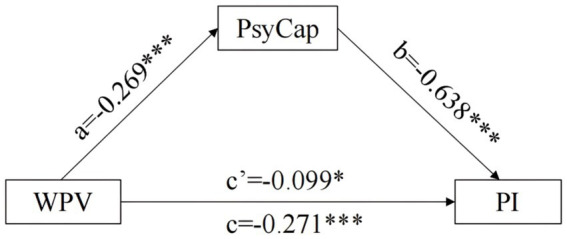
Mediation model of psychological capital. ^*^p < 0.05;^**^p < 0.01; ^***^p < 0.001. PsyCap is positive psychological capital; WPV is workplace violence; PI is the professional identity of nursing interns.

The findings indicated that after controlling for gender, age, monthly household income, educational level, and internship duration, WPV significantly and negatively predicted PsyCap (*a* = −0.269, SE = 0.060, *p* < 0.001; Table 3). When both WPV and PsyCap were included in the regression equation, they significantly predicted professional identity (*c* = −0.099, SE = 0.045, *p* < 0.05; *b* = 0.638, SE = 0.042, *p* < 0.001), with detailed results presented in Supplementary Table 3. The bias-corrected percentile Bootstrap method revealed that PsyCap significantly mediated the relationship between WPV and professional identity (*ab* = −0.171, BootSE = 0.050, 95%CI[−0.286, −0.086]), accounting for 63.10% of the total effect (*ab*/(*ab* + *c*) = 63.10%). This indicated that PsyCap partially mediated the relationship between WPV and professional identity, as illustrated in Figure 2.

**Table 3:** Regression analysis between variables.

Outcome variables	Predictors	Overall fitted index		Regression coefficient significance		
		*R*	*R^2^*	*F*	*β*	*t*
PsyCap	WPV	0.363	0.131	7.413^***^	−0.269	−4.512^***^
PI	WPV	0.739	0.545	50.225^***^	0.099	2.177^*^
PI	PsyCap				0.638	15.090^***^

PsyCap is positive psychological capital; WPV is workplace violence; PI is the professional identity of nurse students. ^*^p < 0.05;^**^p < 0.01; ^***^p < 0.001.

## 4 Discussion

Clinical internships for nursing interns in China are often conducted in high-intensity hospital environments, representing a critical stage for the formation and development of their professional identity. However, the current curriculum tends to prioritize the assessment of clinical skills and knowledge, while psychosocial support for interns is often insufficient (26, 27). Specifically, Tu et al. (26) noted that while interns commonly face occupational stress and positive coping capacity serves as a key resource for mitigating stress and maintaining professional identity, systematic training in this area is often lacking in current educational practices. Concurrently, there is an urgent demand among nursing students for guidance aimed at enhancing career planning, yet existing curricula appear to provide insufficient coverage in this regard (27). This context underscores the urgency and value of conducting focused research on key psychosocial variables such as professional identity, workplace violence response, and psychological capital. This study examined latent profiles of professional identity and their associations with WPV and PsyCap among 301 nursing interns from 9 provinces in China. All research hypotheses were supported.

First, latent profile analysis identified three latent categories of professional identity among nursing interns: “Low Identity” (10.3%), “Moderate Identity” (47.2%), and “High identity-Low autonomy” (42.5%). These findings are consistent with Hypothesis 1, pointing to significant group heterogeneity in their professional identity. The results are generally consistent with trends reported by other Chinese scholars (28). The profiles revealed discernible disparities in identity levels and psychological traits. The low identity group showed the poorest scores across all professional identity dimensions and lower levels of PsyCap, which might suggest concurrent deficiencies in both their professional cognition and psychological resources. The moderate identity group, constituting the largest proportion, might represent interns are in an intermediate stage of career identity formation. They appeared to have established a basic professional cognitive framework but had not yet developed a stable, internalized value judgment system. And they might be particularly susceptible to external influences in clinical settings, such as work conflicts and interpersonal evaluations (29). The high identity-Low autonomy group displayed strong professional value and commitment to nursing alongside perceived limited autonomy in career choice. According to self-determination theory (SDT), autonomy is an individual’s sense of control and freedom in decision-making (30). In light of this theory, one possible interpretation is that nursing interns might largely internalize their professional identity while concurrently experiencing constraints regarding future career choices. This pattern could be associated with nursing students’ still-developing professional knowledge and self-awareness (27), and might also reflect potential constraints posed by current standardized clinical training (31). This classification could have theoretical significance and might offer implications for nursing education and practice. It implies that future nursing education might could consider transitioning from holistic intervention strategies to more precise educational guidance tailored to interns with different types of professional identity profiles.

Second, the findings revealed a significant negative correlation between WPV and professional identity (*r* = −0.31, *p* < 0.001), which is consistent with Hypothesis 2. Multiclass logistic regression further indicated that emotional abuse, threats, and WPV collectively were significant risk factors for nursing interns being classified into the low identity group. This finding is generally consistent with the research by Kim et al. (32), which suggests that WPV, as a negative occupational experience, may be associated with a reduced nursing interns’ sense of belonging and value recognition toward the nursing profession. From the perspective of social identity theory (12), violent incidents might be interpreted by nursing interns as a devaluation of their professional group’s values. Such an interpretation could undermine the foundation of their professional identity. It is noteworthy that among different types of violence, non-physical violence, such as emotional abuse and threats, may pose particularly severe challenges due to its high frequency and concealed nature. This may manifest as damage to professional standing, insults to personal dignity, and threats to workplace security, potentially exerting a cumulative negative influence on professional identity over time (33). Based on the aforementioned research, WPV incidents may not only be detrimental to nursing interns’ mental health but are also associated with a diminished sense of professional value, possibly due to perceived inadequate occupational rewards (34). These effects could contribute to a decline in professional identity and foster intentions to leave their positions (35). This negative association appears particularly pronounced among nursing interns with weaker professional identity. Although causal pathways cannot be confirmed due to the cross-sectional design, preventing and controlling WPV, especially covert emotional abuse, remains crucial for safeguarding interns’ professional identity. It might be beneficial to incorporate WPV response training into clinical education programs, along with communication skills development, to help create safer clinical learning environments.

Third, the findings indicated that PsyCap partially mediated the relationship between WPV and professional identity among nursing interns, supporting Hypothesis 3. The indirect effect was significant (*ab* = −0.171, BootSE = 0.050, 95%CI [−0.286, −0.086]), accounting for 63.10% of the total effect. This suggests that PsyCap may play a protective role in buffering the negative association of WPV (13). This finding aligns with social identity theory. Specifically, as a positive psychological resource for coping with stress, PsyCap may help buffer against potential threats to professional identity damage when interns experience violence related to their professional role (36). This result is consistent with findings reported across various populations (15, 37), providing further support for the potential role of PsyCap in fostering professional identity. The self-efficacy and hope dimensions appeared particularly prominent in the high identity-low autonomy group. These dimensions might empower interns to actively assess stressful events when confronting complex clinical care scenarios, potentially reframing challenges as skill-building opportunities (38). Furthermore, a sense of hope might motivate nursing interns to proactively seek career development resources. For instance, they may participate in continuing education or clinical research (39). This proactive behavior may contribute to maintaining their adaptability and psychological resilience regarding violent incidents, thereby helping to attenuate the negative influence. In contrast, insufficient PsyCap resources might render nursing interns’ positive expectations for career development more vulnerable to erosion from repeated exposure to violent acts like emotional abuse. This association appears especially pronounced in groups with lower professional identity and could potentially be linked to some interns questioning their career choices (2). Therefore, incorporating PsyCap cultivation alongside the integration of violence prevention into clinical teaching management could be a beneficial approach. Such an approach has the potential to enhance interns’ stress coping abilities and professional identity levels.

In summary, LPA analysis revealed significant heterogeneity in nursing interns’ professional identity. Furthermore, a negative correlation was observed between WPV and professional identity, with PsyCap serving as a partial mediator in this association. Based on these findings, to support the development of professional identity among nursing interns, implementing targeted educational interventions while also addressing WPV prevention and PsyCap cultivation could be beneficial. Specifically, adopting categorized guidance strategies might be useful. For the low identity group, it may be beneficial to focus on professional value guidance and PsyCap cultivation, drawing on validated Psychological Capital Intervention (PCI) models (40). Given the substantial curriculum load, interventions could be integrated into pre-internship preparation or embedded into clinical practice through brief resilience training, mindfulness training, and mentorship programs (41). Furthermore, psychological support resources such as counseling and team-building activities can be offered, and fragmented time could be utilized to provide psychological and skills support (42). For the moderate identity group, fostering a stable practical environment along with positive feedback mechanisms might support their professional identity. One potential strategy involves implementing regular, competency-based feedback sessions (43). Such sessions may assist nursing interns in consciously connecting positive clinical experiences with their personal professional role growth, potentially bolstering professional identity and buffering against the impact of external environmental factors. For the high identity-low autonomy group, interventions emphasizing autonomy support could be particularly relevant. Methods such as case-based learning and autonomy-supportive teaching, which have been widely applied and validated in medical and nursing education, have been associated with improvements in critical thinking, clinical reasoning, competence perception, and decision-making confidence (44, 45). Thus, utilizing these structured, active-participation strategies may offer benefits over conventional teaching for this subgroup. These differentiated strategies may help to address the developmental needs of diverse nursing interns, potentially fostering positive growth in their professional identity.

### 4.1 Limitations

First, the cross-sectional design precludes causal inference and does not allow examination of dynamic changes in nursing interns’ professional identity, PsyCap, and WPV over the course of internship training. Second, convenience sampling may have limited representativeness. Although participants were recruited from nine provincial regions, the uneven geographical distribution and female-dominant sample may restrict generalizability. Excluding interns with severe psychological distress may introduce selection bias, underestimate workplace violence exposure and deficits in psychological capital, and reduce the external applicability of the findings to vulnerable groups. Third, important contextual factors (e.g., clinical department differences, supervisor support, and exposure intensity) were not fully assessed, which may have influenced the accuracy of mediation estimates. For instance, supervisor support may buffer the psychological impact of WPV and affect PsyCap, thereby shaping indirect pathways. Fourth, WPV experiences may be underreported due to normalization of violence in clinical settings, and self-report measures are subject to recall and social desirability biases. Although Harman’s one-factor test was performed, future research should employ more rigorous methods (e.g., CFA marker techniques (46)) to control for common method bias. Finally, cultural characteristics of the Chinese nursing education context may limit transferability to other settings. Future research should adopt longitudinal designs to clarify temporal and causal relationships. More diverse and balanced samples across regions and genders are needed to improve representativeness. Future studies should also incorporate key contextual factors (e.g., department characteristics and supervisory support) and use multi-source WPV assessments (e.g., supervisor reports and institutional records) to reduce reporting bias and enhance generalizability.

## 5 Conclusion

This study employed LPA to reveal the heterogeneous characteristics of professional identity among nursing interns, identifying three latent categories: “Low Identity” group, “Moderate Identity” group, and “High identity-Low autonomy” group. Findings indicate a significant negative correlation between WPV and professional identity, with PsyCap partially mediating this relationship. Based on these results, it may be beneficial for nursing educators and administrators to consider adapting clinical education to consider adapting psychological capital development while improving early intervention mechanisms for workplace violence. Furthermore, the study provides evidence-based support for policymakers to formulate targeted strategies. These measures are crucial for enhancing professional identity and WPV coping capacity, and they also provide a foundation for customized talent cultivation policies.

## Data Availability

The raw data supporting the conclusions of this article will be made available by the authors, without undue reservation.

## References

[1] WHO. World Health Organization. State of the world’s nursing 2025: investing in education, leadership and service delivery. (2025). Available online at: https://www.who.int/publications/i/item/9789240110236/ (Accessed February 14, 2026).

[2] Zheng X Song J Shi X Lu G Qu Z Lu X . The effect of professional identity on new nurses’ turnover intention: the mediating role of psychological capital and achievement motivation. BMC Psychol. (2025) 13:924. doi: 10.1186/s40359-025-03295-7, 40820146 PMC12360020

[3] Haslam SA Powell C Turner J. Social identity, self-categorization, and work motivation: rethinking the contribution of the group to positive and sustainable organisational outcomes. Appl Psychol. (2000) 49:319–39. doi: 10.1111/1464-0597.00018

[4] Wei L Zhou S Hu S Zhou Z Chen J. Influences of nursing students’ career planning, internship experience, and other factors on professional identity. Nurse Educ Today. (2021) 99:104781. doi: 10.1016/j.nedt.2021.104781, 33530029

[5] Mousazadeh S Yektatalab S Momennasab M Parvizy S. Job satisfaction challenges of nurses in the intensive care unit: a qualitative study. RMHP. (2019) 12:233–42. doi: 10.2147/RMHP.S218112, 32009822 PMC6859118

[6] Kim J Chae D Yoo JY. Reasons behind generation Z nursing students’ intentions to leave their profession: a cross-sectional study. Inquiry. (2021) 58:0046958021999928. doi: 10.1177/0046958021999928, 33660536 PMC7940812

[7] Zhang M Wang Q Chen Y He M Zhou W Yao Z . Internship and postgraduate entrance examination: a qualitative study on the psychological experience of undergraduate nursing students under dual pressure in China. Heliyon. (2024) 10:e37644. doi: 10.1016/j.heliyon.2024.e37644, 39309269 PMC11413662

[8] Zhou Y Yao Q Li W Jian Q Luo Z. Meta-analysis of the incidence and influencing factors of workplace violence among nursing interns. Nurs Pract Res. (2023) 20:3413–20. doi: 10.3969/j.issn.1672-9676.2023.22.017

[9] Pagnucci N Ottonello G Capponi D Catania G Zanini M Aleo G . Predictors of events of violence or aggression against nurses in the workplace: a scoping review. J Nurs Manag. (2022) 30:1724–49. doi: 10.1111/jonm.13635, 35420236 PMC9796891

[10] Selye H. A syndrome produced by diverse nocuous agents. JNP. (1998) 10:230a–1a. doi: 10.1176/jnp.10.2.230a, 9722327

[11] Shi L Li G Hao J Wang W Chen W Liu S . Psychological depletion in physicians and nurses exposed to workplace violence: a cross-sectional study using propensity score analysis. Int J Nurs Stud. (2020) 103:103493. doi: 10.1016/j.ijnurstu.2019.103493, 31884332

[12] Brown R. The social identity approach: appraising the Tajfellian legacy. Br J Soc Psychol. (2020) 59:5–25. doi: 10.1111/bjso.12349, 31691319

[13] Yuan Z Zhang X Wang F Jin M Teng M He H . Levels of psychological capital among nurses: a systematic review and meta-analysis. Int Nurs Rev. (2023) 70:89–96. doi: 10.1111/inr.12803, 36205604

[14] Del Prato D Bankert E Grust P Joseph J. Transforming nursing education: a review of stressors and strategies that support students’ professional socialization. Adv Med Educ Pract. (2011) 2:109–16. doi: 10.2147/AMEP.S18359, 23745082 PMC3661250

[15] Chang T Jiang X Wei J Zhao J Li Z Li H. Mediating effects of psychological capital on the relationship between workplace violence and professional identity among nurses working in Chinese public psychiatric hospitals: a cross-sectional study. BMJ Open. (2023) 13:e065037. doi: 10.1136/bmjopen-2022-065037, 36599638 PMC9815003

[16] Williams GA Kibowski F. "Latent class analysis and latent profile analysis" In: Jason LA Glenwick DS, editors. Handbook of methodological approaches to community-based research: qualitative, quantitative, and mixed methods. New York: Book, Oxford University Press (2016)

[17] Yu LJ Wang WJ Xing JX. Analysis of latent potential profiles and influencing factors of intern nursing students’ professional identity in Shandong Province [in Chinese]. Zhonghua Lao Dong Wei Sheng Zhi Ye Bing Za Zhi. (2024) 42:517–22. doi: 10.3760/cma.j.cn121094-20231116-00116, 39075006

[18] Wang M. Latent variable modeling and Mplus applications: advanced edition [in Chinese]. China: Book, Chongqing University Press (2018).

[19] Schat ACH Kelloway EK. Reducing the adverse consequences of workplace aggression and violence: the buffering effects of organizational support. J Occup Health Psychol. (2003) 8:110–22. doi: 10.1037/1076-8998.8.2.110, 12703877

[20] Wang P. Study on medical workplace violence and theory model [in Chinese]. Sichuan University (2006). Available from: https://med.wanfangdata.com.cn/Paper/Detail?id=DegreePaper_Y995339 (Accessed February 24, 2026).,

[21] Hao. A study of self-education model to enhance nursing students’ professional identity and professional self-efficacy [in Chinese]. Shanghai: Second Military Medical University (2011).

[22] Zhang K Zhang S Dong Y. Positive psychological capital: Measurement and its relationship to mental health. Psychological and Behavioral Research. Studies of Psychology and Behavior. (2010) 8:58. Available online at: https://psybeh.tjnu.edu.cn/EN/abstract/abstract930.shtml (Accessed February 24, 2026).

[23] Luthans F Avolio BJ Avey JB Norman SM. Positive psychological capital: measurement and relationship with performance and satisfaction. Pers Psychol. (2007) 60:541–72. doi: 10.1111/j.1744-6570.2007.00083.x

[24] Wen Z Ye B. Mediation effects analysis: methodology and model development [in Chinese]. Adv Psychol Sci. (2014) 22:731. doi: 10.3724/SP.J.1042.2014.00731

[25] Kim S-Y. Determining the number of latent classes in single- and multi-phase growth mixture models. Struct Equ Modeling. (2014) 21:263–79. doi: 10.1080/10705511.2014.882690, 24729675 PMC3979564

[26] Tu H Liu J Li F Lin T Jin P Li P . The mediating effect of coping on perceived stress and professional identity among nursing interns: a cross-sectional study conducted in a medical university in China. BMC Psychol. (2024) 12:682. doi: 10.1186/s40359-024-02208-4, 39574160 PMC11583482

[27] Zhou H Huang L Fu Z Li L Huang Z Zhang J . Factors associated with career decision-making difficulties among undergraduate nursing students: a latent profile analysis. Front Med. (2026) 12:1644508. doi: 10.3389/fmed.2025.1644508, 41567675 PMC12816191

[28] Li F Ning L Li S Fu Y Wang Y Deng Q . Latent profiles of nursing students’ professional identity and their relationship with stress and coping styles during clinical practicum. Nurse Educ Pract. (2023) 73:103840. doi: 10.1016/j.nepr.2023.103840, 37972464

[29] Traynor M Buus N. Professional identity in nursing: UK students’ explanations for poor standards of care. Soc Sci Med. (2016) 166:186–94. doi: 10.1016/j.socscimed.2016.08.024, 27567092

[30] McAnally K Hagger MS. Self-determination theory and workplace outcomes: a conceptual review and future research directions. Behav Sci (Basel). (2024) 14:428. doi: 10.3390/bs14060428, 38920760 PMC11200516

[31] Perry C Henderson A Grealish L. The behaviours of nurses that increase student accountability for learning in clinical practice: an integrative review. Nurse Educ Today. (2018) 65:177–86. doi: 10.1016/j.nedt.2018.02.029, 29587209

[32] Kim S Lynn MR Baernholdt M Kitzmiller R Jones CB. How does workplace violence–reporting culture affect workplace violence, nurse burnout, and patient safety? J Nurs Care Qual. (2023) 38:11. doi: 10.1097/NCQ.0000000000000641, 36409656

[33] Wang J Lyu H Xing X. Prevalence and risk factors of workplace violence among medical workers in China: a cross-sectional study. Med Soc. (2021) 34:29–33. doi: 10.13723/j.yxysh.2021.08.006

[34] Dafny HA McCloud C Pearson V Brown S Phillips C Waheed N . Nursing students’ experience of workplace violence in clinical practice: a qualitative systematic review. J Clin Nurs. (2023) 32:6136–64. doi: 10.1111/jocn.16746, 37166364

[35] Hallett N Gayton A Dickenson R Franckel M Dickens GL. Student nurses’ experiences of workplace violence: a mixed methods systematic review and meta-analysis. Nurse Educ Today. (2023) 128:105845. doi: 10.1016/j.nedt.2023.105845, 37300926

[36] Parviniannasab AM Bijani M Dehghani A. The mediating role of psychological capital in relations between spiritual well-being and mental health among nursing students. BMC Psychol. (2022) 10:230. doi: 10.1186/s40359-022-00935-0, 36184628 PMC9526786

[37] Deng J Xu Y Li Q Yang W Deng H. The relationship between psychological capital, patient’s contempt, and professional identity among general practitioners during COVID-19 in Chongqing, China. PLoS One. (2023) 18:e0287462. doi: 10.1371/journal.pone.0287462, 37812597 PMC10561861

[38] Huang L Zhang X Wang F Zhang S Chang X Chu Y . The relationship between reflective ability and professional identity: the mediating effect of self-directed learning and self-efficacy for junior clinical nurses. BMC Nurs. (2024) 23:858. doi: 10.1186/s12912-024-02534-3, 39587597 PMC11590490

[39] Price S Reichert C. The importance of continuing professional development to career satisfaction and patient care: meeting the needs of novice to mid- to late-career nurses throughout their career span. Adm Sci. (2017) 7:17. doi: 10.3390/admsci7020017

[40] Mahmoud MA Abdelrahim SM Hamed AEM Zoromba MA Mohamed AF. Fortifying the nursing workforce: nursing-led psychological capital interventions to advance resilience, occupational hardiness, and engagement: a randomized controlled trial. Int Nurs Rev. (2025) 72:e70094. doi: 10.1111/inr.70094, 40810664

[41] Shi Z Dai X Cai Z OuYang Z Sun C Sun X. The influence of psychological capital on nursing interns’ role breadth self-efficacy and vertical violence: based on potential profile analysis [in Chinese]. Health Voc Educ. (2025) 43:144–9. doi: 10.20037/j.issn.1671-1246.2025.18.38

[42] Qiu S Lu D. Latent profile analysis of psychological capital and its influencing factors among nurses in a tertiary hospital in Guangzhou [in Chinese]. Psychol Mon. (2025) 20:202–5. doi: 10.19738/j.cnki.psy.2025.22.054

[43] Sun Y Li X Liu H Li Y Gui J Zhang X . The effectiveness of using situational awareness and case-based seminars in a comprehensive nursing skill practice course for undergraduate nursing students: a quasi-experimental study. BMC Med Educ. (2024) 24:118. doi: 10.1186/s12909-024-05104-y, 38321409 PMC10848502

[44] Varma B Karuveettil V Fernandez R Halcomb E Rolls K Kumar SV . Effectiveness of case-based learning in comparison to alternate learning methods on learning competencies and student satisfaction among healthcare professional students: a systematic review. J Educ Health Promot. (2025) 14:76. doi: 10.4103/jehp.jehp_510_24, 40144176 PMC11940068

[45] Neufeld A. Autonomy-supportive teaching in medicine: from motivational theory to educational practice. MedEdPublish. (2021) 10. doi: 10.15694/mep.2021.000117.1, 38486596 PMC10939533

[46] Richardson HA Simmering MJ Sturman MC. A tale of three perspectives: examining post hoc statistical techniques for detection and correction of common method variance. Organ Res Methods. (2009) 12:762–800. doi: 10.1177/1094428109332834

