# Supplementary material for: Workplace violence, psychological capital, and professional identity among Chinese nursing interns: a latent profile and mediation analysis
Source: Front Public Health. 2026 Mar 3;14:1791963. doi: 10.3389/fpubh.2026.1791963 (PMC12992273; doi:10.3389/fpubh.2026.1791963)
Supplement: Supplementary file 1 [file Data_Sheet_1.docx]

**Supplementary Table 1** Correlations among study variables.

| **Variables** | **Mean** | **SD** | **1.** | **2.** | **3.** |
| --- | --- | --- | --- | --- | --- |
| 1.WPV | 1.09 | 2.15 | 1 |  |  |
| 2.PsyCap | 118.96 | 21.93 | -0.26^***^ | 1 |  |
| 3.PI | 56.71 | 12.52 | -0.31^***^ | 0.70^***^ | 1 |

Note:^*^*P* < 0.05; ^**^*P* < 0.01; ^***^*P* < 0.001. SD = standard deviation. PsyCap is positive psychological capital; WPV is workplace violence; PI is the professional identity of nursing interns.

**Supplementary Table 2** Characteristics and differences of nursing interns in WPV and PsyCap across professional identity profiles.

| **Variable** | **Overall**  **(n=301)** | **Low**  **Identity**  **(n=31)** | **Moderate**  **Identity**  **(n=142)** | **High identity**  **-Low autonomy**  **(n=128)** | **F**  **(df_1_=2,**  **df_2_=298)** | **η^2^** |
| --- | --- | --- | --- | --- | --- | --- |
| Psychological capital (total) | 118.97±21.89 | 96.71±4.80 | 109.85±1.15 | 134.48±1.53 | 100.692^***^ | 0.400 |
| Self efficacy | 33.12±7.34 | 25.58±1.74 | 30.11±0.37 | 38.30±0.50 | 99.852^***^ | 0.401 |
| Tenacity | 28.06±6.36 | 23.23±1.50 | 27.46±0.41 | 29.91±0.58 | 16.520^***^ | 0.100 |
| Hope | 28.43±5.49 | 24.39±1.25 | 25.92±0.30 | 32.20±0.40 | 85.383^***^ | 0.356 |
| Optimistic | 29.35±6.33 | 23.51±1.34 | 26.37±0.35 | 34.08±0.41 | 112.923^***^ | 0.431 |
| Psychological capital (total) | 118.97±21.89 | 96.71±4.80 | 109.85±1.15 | 134.48±1.53 | 100.692^***^ | 0.400 |
| Bodily assault | 0.12±0.45 | 0.19±0.12 | 0.14±0.04 | 0.08±0.03 | 1.141 | 0.008 |
| Emotional abuse | 0.58±0.93 | 1.45±0.21 | 0.65±0.08 | 0.3±0.05 | 22.234^***^ | 0.130 |
| Threaten and intimidate | 0.16±0.53 | 0.42±0.15 | 0.18±0.05 | 0.08±0.03 | 5.481^***^ | 0.035 |
| Verbal harassment | 0.16±0.54 | 0.19±0.09 | 0.23±0.05 | 0.08±0.03 | 2.633 | 0.017 |
| Physical harassment | 0.07±0.37 | 0.13±0.09 | 0.06±0.03 | 0.05±0.03 | 0.492 | 0.003 |
| Workplace violence (total) | 1.09±2.15 | 2.39±0.49 | 1.26±0.20 | 0.59±0.13 | 10.114^***^ | 0.06 |

Note:^*^*P* < 0.05; ^**^ *P* < 0.01; ^***^ *P* < 0.001.

**Supplementary Table 3**  Analysis of the effects of WPV on professional identity.

| **Effect** | **Ways** | **Standardized effect value** | **Standard errors** | ***t*** | ***P*** | ***95%CI*** |
| --- | --- | --- | --- | --- | --- | --- |
| Direct effect | WPV→PI | -0.099 | 0.045 | -2.226 | <0.05 | -0.187~ -0.012 |
| Indirect effect | WPV→PI | -0.171 | 0.050 | - | - | -0.286 ~ -0.086 |
| Total effect | WPV→PI | -0.271 | 0.057 | -4.713 | <0.001 | -0.384 ~ -0.158 |

Note: ^*^*P* < 0.05;*^**^ P* < 0.01; ^***^ *P* < 0.001. PsyCap is positive psychological capital; WPV is workplace violence; PI is the professional identity of nursing interns.
